# Evaluation of elevated alanine aminotransferase and hepatitis B virus DNA in healthy seronegative blood donors

**DOI:** 10.1186/1756-0500-5-272

**Published:** 2012-06-07

**Authors:** Natasha Ali, Bushra Moiz, Tariq Moatter

**Affiliations:** 1 Department of Pathology and Microbiology, The Aga Khan University Hospital, P.O Box 3500, Stadium Road, Karachi, 74800, Pakistan

**Keywords:** ALT, Hepatitis B, Blood donors

## Abstract

**Background:**

Serum alanine transaminase (ALT) has been used as a surrogate marker for detection of hepatitis B and C in blood donors in Pakistan since 1985. Since the introduction of more sensitive assays the value of ALT became questionable but it was still used with subsequent wastage of blood units with raised ALT.

**Findings:**

We conducted a study for a period of one year to evaluate the usefulness of ALT. During the study period, 25117 subjects donated blood. Eight hundred and seventy two donors (3.4%) were positive for one or more serological tests. ALT of all donors ranged from 0–1501 U/L (Mean ± SD; 33.4 ± 25.45U/L). The donors seronegative for all disease markers were 24245 (96.6%). Of these, 21164 (84.2%) donors had their ALT within reference range while 2874 (11.4%) and 207 (0.8%) of donors had minimal and markedly elevated results respectively. Six hundred and twenty one blood units (including red cells, platelets and plasma) were discarded based on elevated ALT results alone at a cost of $39,123. Two hundred seronegative blood donors with normal ALT, minimally and markedly elevated ALT levels, were selected randomly and evaluated for hepatitis B deoxynucleic acid by individual PCR. None of the donors was found to be reactive.

**Conclusion:**

This work did not support a positive association between hepatitis B virus nucleic acid and elevated ALT in healthy serologically negative blood donors.

## Findings

Hepatitis B virus (HBV) infection is a major health problem with 400 million chronically infected subjects in the world [1]. Serum alanine aminotransferase (ALT) is most frequently used screening test in evaluation of liver damage. It has also been used historically as a surrogate marker in blood donors [2]. Previous studies have shown that alcoholism and obesity are the most common causes of elevated ALT in blood donors [3]. Since newer, sensitive techniques like fourth generation ELISA became available [4], the value of using ALT as a surrogate marker was reconsidered. The cost of ALT per sample is $3 and the expense of discarding a single blood product with elevated ALT is approximately $63 in our setting. Therefore this study aimed to correlate the association of hepatitis B virus deoxyribonucleic acid with serum alanine transaminase in healthy seronegative blood donors.

We enrolled healthy non-commercial blood donors after taking written informed consent between the ages of 17–65 years after approval from institutional ethical review committee of The Aga Khan University. The study was conducted for a period of one year from November 2006 to October 2007. Five ml of blood in a gel tube (BD vacutainer) was collected from the donated blood bag. Tubes were centrifuged for 5 minutes at 2500 g. Separated sera were transferred to eppendorf tubes and transported to molecular research laboratory and samples were kept frozen at -80°C till testing which was done in six weeks time. All donations were screened simultaneously for anti- HIV I and II, anti HCV, HBsAg, syphilis and malaria. Viral serology was performed via third generation CA (Vitros Eci, Johnson and Johnson, Ortho Clinical and Diagnostic, NY, USA) while VDRL (VDRL Carbon antigen Plasmatec Laboratory products - RPR kit) and ICT malaria tests (Now malaria; Binax incorporated, USA) were done manually for screening syphilis and malaria respectively. Each donation was also tested for serum ALT by kinetic method at a reaction wavelength of 340 nm at a temperature of 25°C (Beckman Synchron; CX7, USA). As per institutional policy, all blood bags which were reactive for one or more disease markers and or two times the upper limit of ALT were incinerated. Donors were grouped into three groups according to ALT levels: A: donors within reference range, B: minimally elevated and group C with markedly raised levels. Reference ranges for ALT established in our lab are 3-33U/L for females and 0-55U/L for males. Markedly elevated ALT was defined as results greater than twice the upper limit of reference range (≥110 U/l for males and ≥66 U/l for females). ALT within these two limits was considered as minimal elevations (56–109 U/l for males and 34–65 U/l for females). Within these three groups, 200 seronegative blood donors were randomly selected (group A, n = 88, group B, n = 12 and group C, n = 100) and tested for hepatitis B virus deoxyribonucleic acid to exclude the possibility of occult hepatitis B infection. These donors were seronegative for all viral markers with normal or elevated ALT levels. The test was performed utilizing AJ ROBOSCREEN; (Real time polymerase chain reaction) which has an analytic sensitivity of 50 IU/ml.

During the study period, 25117 subjects donated blood. There were 23685 (94.2%) males and 1432 (5.6%) females with age ranging from 17 to 65 years (median ± SD 27.7 ± 7.621). There were 872 donors (3.4%) who had one or more abnormal screening results. A total of n = 908 abnormal tests were detected. There were 84.2% donors (n = 21164) who had ALT within reference range while minimal and markedly elevated ALT were seen in 11.4% (n = 2874) and 0.8% (n = 207) subjects. Out of n = 908 abnormal tests, donors with elevated ALT and Hepatitis B surface antigen positivity were n = 250 (1.0%) which were excluded from the final analysis.

**Figure 1:**
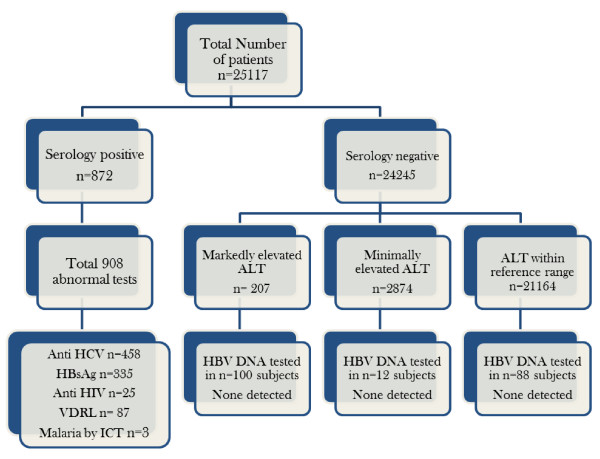
**legend: This figure shows the breakdown of 200 randomly selected seronegative healthy blood donors with markedly elevated, minimally elevated and normal ALT levels.** Hepatitis B deoxyribonucleic acid by RT-PCR was performed in all donors. None of the donors tested positive showing no correlation with elevated ALT and occult hepatitis B infection.

Real Time Polymerase chain reaction for detection of hepatitis B virus deoxyribonucleic acid was performed in 200 randomly selected seronegative donors from groups A, B and C. None of the donors screened positive for hepatitis B virus infection (Figure 1).

Blood banks all over the world have replaced ALT testing as a surrogate marker in blood donors with newer sensitive tests [5]. We performed hepatitis B virus DNA by RT-PCR on seronegative blood donors and none was found to be reactive in all three groups. Since the establishment of blood bank in our laboratory which was in the year 1985, we have been discarding blood units with elevated ALT. With the availability of real time polymerase chain reaction we evaluated the true value of ALT testing in identifying hepatitis B virus infection in our blood donors.

We bleed approximately 25,000 donors annually and most of the blood units collected are from exchange donors. Voluntary donations contribute to 11 % of the above mentioned figure therefore discarding blood units with elevated ALT in this situation results in its wastage. A single collected unit is further processed into packed red cells, platelets and plasma. We found n = 207 blood donors/units with markedly elevated ALT levels and discarding these units meant actually discarding 621 blood products. The cost of performing ALT in 2006 was $3 while the cost of incinerating a blood product is $50. Serological testing was $10 per bag. Therefore cost of discarding these many donations was $ 39,123. We found that ALT testing did not assist in identifying window period donations/occult hepatitis B infection. After this study, we initiated Nucleic Acid Testing (NAT) of our blood donors and ALT testing was discontinued.

Further large scale studies are warranted to explore the association, if any between hepatitis B infection and serum ALT. Our preliminary results showed no such correlation between the two.

## Abbreviations

HBV: Hepatitis B Virus; HCV: Hepatitis C Virus; ALT: Alanine aminotransferase; ELISA: Enzyme linked immunosorbent assay.

## Competing interests

The authors declare that they have no competing interests.

## Authors’ contributions

NA collected samples, drafted the manuscript and research grant. NA also participated in molecular studies and statistical analysis, BM contributed to molecular studies, statistical analysis and drafting of manuscript, TM performed the molecular tests and contributed to manuscript drafting. All authors read and approved the final manuscript.
